# Leaf side determines the relative importance of dispersal versus host filtering in the phyllosphere microbiome

**DOI:** 10.1128/mbio.01111-23

**Published:** 2023-07-12

**Authors:** Wenke Smets, Mason K. Chock, Corinne M. Walsh, Caihong Qiu Vanderburgh, Ethan Kau, Steven E. Lindow, Noah Fierer, Britt Koskella

**Affiliations:** 1 Department of Integrative Biology, University of California, Berkeley, California, USA; 2 Department of Bioscience Engineering, University of Antwerp, Antwerpen, Belgium; 3 Cooperative Institute for Research in Environmental Sciences, University of Colorado, Boulder, Colorado, USA; 4 Department of Ecology and Evolutionary Biology, University of Colorado, Boulder, Colorado, USA; 5 Department of Plant and Microbial Biology, University of California, Berkeley, California, USA; 6 Chan Zuckerberg Biohub, San Francisco, California, USA; University of Toronto, Toronto, Ontario, Canada

**Keywords:** phyllosphere, microbial ecology, leaf surface

## Abstract

Leaves harbor distinct microbial communities that can have an important impact on plant health and microbial ecosystems worldwide. Nevertheless, the ecological processes that shape the composition of leaf microbial communities remain unclear, with previous studies reporting contradictory results regarding the importance of bacterial dispersal versus host selection. This discrepancy could be driven in part because leaf microbiome studies typically consider the upper and lower leaf surfaces as a single entity despite these habitats possessing considerable anatomical differences. We characterized the composition of bacterial phyllosphere communities from the upper and lower leaf surfaces across 24 plant species. Leaf surface pH and stomatal density were found to shape phyllosphere community composition, and the underside of leaves had lower richness and higher abundances of core community members than upper leaf surfaces. We found fewer endemic bacteria on the upper leaf surfaces, suggesting that dispersal is more important in shaping these communities, with host selection being a more important force in microbiome assembly on lower leaf surfaces. Our study illustrates how changing the scale in which we observe microbial communities can impact our ability to resolve and predict microbial community assembly patterns on leaf surfaces.

**IMPORTANCE:**

Leaves can harbor hundreds of different bacterial species that form unique communities for every plant species. Bacterial communities on leaves are really important because they can, for example, protect their host against plant diseases. Usually, bacteria from the whole leaf are considered when trying to understand these communities; however, this study shows that the upper and lower sides of a leaf have a very different impact on how these communities are shaped. It seems that the bacteria on the lower leaf side are more closely associated with the plant host, and communities on the upper leaf side are more impacted by immigrating bacteria. This can be really important when we want to treat, for example, crops in the field with beneficial bacteria or when trying to understand host-microbe interactions on the leaves.

## INTRODUCTION

Phyllosphere (leaf-associated) bacterial communities are increasingly recognized for their role in plant health, modulating chemical emissions of plants, providing nutrients to plants (e.g., nitrogen fixation), and protecting against plant pathogens (1 - 3). Leaves typically harbor bacterial populations ranging in size from 10^6^ to 10^7^ cells/cm^2^ leaf (4). Above-ground plant biomass (the phyllosphere) has been estimated to comprise 60% of biomass across all taxa on Earth and contributes substantially to global biogeochemical cycles and ecosystems (5). The bacterial communities found on leaves are distinct from those in other environmental habitats or host organisms and also differ substantially in composition among host plant species. Some of the distinctiveness of epiphytic bacterial communities can be attributed to abiotic factors, including UV exposure (6, 7), temperature (8), and the differential availability of water (9, 10). Likewise, the composition of leaf-associated microbial communities can be driven by unique biotic factors, including leaf structure and physiology (11, 12), and interactions among microbes, including with bacteriophage (13, 14). In addition to these deterministic processes, phyllosphere communities—like all microbiomes —are subject to stochasticity during assembly due to differential exposure to immigrant inoculum sources (15 - 18) and the random nature of arrival time and the order of arrival of dispersing microbes (17, 19).

Although phyllosphere community variation has been attributed to differences in plant species identity and phylogeny (20 - 22), even leaves from the same individual plant, or those from different individual plants of a single plant species at a given sampling site, can harbor remarkably distinct bacterial communities (23, 24). Results from previous phyllosphere studies show a discrepancy between the relative contribution of location, driven by dispersal (a neutral process), and host plant selection (a deterministic process). These apparent inconsistencies in the importance of deterministic versus neutral processes might be related to the fact that it is often implicitly assumed that the leaf microbiome of an individual plant constitutes a single community.

Leaves of most plant species have distinct anatomies and physiologies between their upper (adaxial) and lower (abaxial) surfaces. We expect that topographic features which differ between the leaf sides will influence microbial community sizes and interactions, as exemplified by bacterial aggregations often observed in grooves between plant cells, or by trichomes, and stomata (25). Topographical features also strongly influence the distribution and movement of water on leaves, thereby influencing bacterial localization and their association with water (10). Environmental stressors typically encountered in the phyllosphere, including UV irradiation, periodic desiccation, and changes in humidity levels (4, 7, 26), are expected to be more intense on the upper than on the lower leaf surface. Moreover, in most plant species, stomatal density is higher on the lower surface of the leaf than on the upper surface (27), possibly contributing to higher local humidity levels due to the trapping of evapotranspired water within the laminar boundary layer surrounding the leaf (28). Besides topographical features, leaf leachates, which are expected to be more abundant on the lower leaf surface due to a thinner cuticle, may play an important role in selecting the microbes that live on the leaf surfaces (29, 30). These species-specific leaf leachates might lead to stronger host “filtering” effects on the lower leaf surfaces compared to the upper leaf surfaces. In contrast, the upper surfaces of leaves are more likely to intercept airborne bacteria than the underside, due to higher rates of deposition of airborne particles on the upper leaf surface (31 - 33). A higher influx of colonizing bacteria decreases the relative strength of host filtering on phyllosphere microbiome composition (18); hence, we might expect stronger host filtering on the lower leaf surface compared to the upper. Although observations comparing bacterial communities of opposite leaf sides are limited, measurements of viable cells and microscopic observations have found higher numbers of bacteria on the lower compared to the upper surface of leaves in sun-exposed peanut plants and in *Arabidopsis* plants grown in the field (34, 35). We hypothesize that the higher humidity and elevated availability of resources make the lower surface of a leaf more conducive to bacterial growth, activity, and interaction with the host than the upper leaf surface. These factors, combined with reduced dispersal of microbes onto lower leaf surfaces, would lead to a stronger signature of host filtering for phyllosphere microbial communities on the lower surface versus the upper surface of leaves.

To test the relative impact of leaf physiology and differential exposure of leaves to bacterial immigration on the microbial community, we sampled replicate leaves from 24 plant species growing in the University of California Botanical Garden at Berkeley, USA. By comparing the composition of epiphytic bacterial communities between the upper and lower surface of leaves, we were able to directly test the hypotheses that (1) the influence of host plant species on leaf microbiome composition is higher on the underside of leaves and (2) upper leaf surfaces harbor more transient taxa that disperse and accumulate via atmospheric deposition, thereby overcoming host filtering effects on community composition.

## MATERIALS AND METHODS

### Sampling location

Plants were sampled from the University of California Botanical Garden at Berkeley, USA (37°52′30″N, 122°14′15″W) over 4 days from 14 April 2021 to 19 April 2021. During this period, the 6-h temperature averages ranged from 8°C to 23°C, the relative humidity average ranged from 47% to 88%, and no precipitation occurred. However, most plants in the botanical garden were irrigated at least once a week. We selected plant species so that our samples would cover a wide range of leaf morphologies and plant growth forms (tree-like, shrub, or herbaceous). Furthermore, the leaves had to be at least 2 months old by the time we sampled, and each sampled plant species was represented by three individuals in the botanical garden. We only sampled leaves that did not touch any soil surface to minimize soil contamination. Due to limits of swab sampling, conifers and plants with very small leaves were not included. In total, we sampled 24 different plant species with three individual plants per species that were sampled randomly over the different days (due to practical reasons four species were represented by only one or two individuals).

### Leaf sampling

Leaves were excised at the petiole with shears sterilized with 70% ethanol, immediately placed in a sterile plastic bag, and processed within 1 h of sampling. To provide a sufficient area for microbial and pH sampling, 1 to 8 leaves, depending on leaf sizes, corresponding to a total leaf area ranging from 94 to 276 cm^2^, were sampled from each individual plant. The coordinates of each sampled plant were obtained using a Garmin eTrex 30 x GPS device.

### Microbial measurements

To sample the microbiome on leaf surfaces, a sterile flock swab (Puritan, 25–3306-H) was dipped in a sterile wash solution (autoclaved 10 mM MgCl_2_ containing 0.002% Tween), and one half of the leaf surface (left of midrib, including half of the midrib) was thoroughly swabbed. Swabs were then placed in sterile, dry 1.5 mL tubes, transported to the lab on ice, and stored at −20°C on the same day. One blank swab, dipped in wash solution and placed directly in a 1.5 mL tube, was included as a control. For each plant sample, the lower and upper sides of the leaf were swabbed separately. For each sampled leaf, the left half of both the lower and upper sides was separately swabbed, allowing pH measurements to be taken of the non-disturbed, right half of each leaf.

### pH measurements

Following swabbing to remove bacteria from the leaf surface, the pH of the upper and lower leaf surface was measured using a sterile cotton swab (Puritan, 806-WC). Briefly, a swab was soaked in a 1.5 mL tube containing 300 µL of sterile deionized H_2_O, and the right halves of the leaves were swabbed with the wetted swab and the swab was then returned to the 1.5 mL tube and the wooden handle excised to ensure that the tube could be closed. The tube was vortexed with the swab for 20 s. The swab was then pressed against the sides of the tube to squeeze the liquid out and discarded. The pH probe (Sartorius, PY-P21) was placed in the small volume in the tube, and a pH meter (Denver instruments, UB-5) was used to measure the pH after readings stabilized within 2 min. Consistency of the leaf surface pH methodology was based on preliminary trials across various plant species. These trials revealed that our chosen methodology yielded pH values that were similar to that obtained by placing a flat-bottom electrode on a wetted leaf for 15 min, a method used for leaf pH measurements previously (36).

### Leaf characteristics

Following the measurement of pH, several leaf characteristics, including stomatal density, leaf hardness, and leaf pubescence, were recorded. Stomatal density was measured using epidermal leaf impressions (37). Temporary stomatal impressions were made by coating unswabbed leaf samples with a layer of clear nail varnish. For each leaf, stomatal slides were taken near the midvein at the base, middle, and tip of each leaf, avoiding the midrib. After drying, impressions were removed, observed, and imaged under a light microscope at 200 X (Olympus, BH-2; Nikon, D200). All stomata in one microscopic field were counted, and stomatal density was determined as the mean of the three leaf locations, after normalizing for the magnification and area examined. Leaf area was measured using an area meter (LI-COR, LI-3100C). Leaf hardness was judged on a qualitative scale of 0 to 3, with zero being soft/thin and four being hard/brittle (38). Lastly, leaf pubescence was assessed based on the presence or absence of leaf hairs visualized under a microscope.

### DNA extraction and sequencing

Microbial swabs were sent on dry ice to the University of Colorado Boulder for DNA extraction and marker gene sequencing. DNA was extracted from the swabs using a tube-based DNeasy Powersoil Pro Kit (Qiagen) to minimize the possibility of well-to-well contamination across these presumably low-biomass leaf surface samples. DNA extractions were performed according to the manufacturer’s protocol with two exceptions: after CD1 addition and before the bead beating step, samples were incubated at 65°C for 10 min, and DNA was eluted with 50 µL C6 solution as opposed to 100 µL.

All leaf swab DNA extractions were PCR amplified targeting the 16S rRNA gene (hypervariable V4–V5 region) using 515f/806r barcoded primers (39, 40). PCR amplification was performed in duplicate for 133 leaf swab samples, 14 DNA extraction blank negative controls, and 13 no-template control (PCR) negative controls. Resulting amplicons were pooled, cleaned, and normalized using SequalPrep Normalization Plates (Thermo Fisher Scientific, Waltham, MA). Pooled samples were sent to CU Anschutz School of Medicine core facility for Next Generation Sequencing on an Illumina MiSeq using 2 × 150 paired end chemistry. All sequences are deposited under project accession number PRJEB55637.

### Bioinformatic analyses

Amplicon Sequence Variants (ASVs) were generated from raw reads using an in-house bioinformatic pipeline based around DADA2 (41) as performed previously (DADA2 version 1.14.1, Fierer Lab DADA2 Pipeline version 0.1.0) (42). Prior to sequence inference, reads were filtered and trimmed using the following settings: truncLen = c(145,150), maxEE = c(2, 2), truncQ = 2, maxN = 0, rm.phix = TRUE. Reads from all samples were pooled for sequence inference using the pool = TRUE parameter of the dada() function. The resulting ASVs were further processed to remove chimeras and assign taxonomy using the DADA2 naïve Bayesian classifier with the SILVA database version 132 (43).

Data were imported in R, and further processing was done in RStudio using R version 3.6.3 mainly using the R packages tidyverse (44) and tidyamplicons (https://github.com/Swittouck/tidyamplicons). Reads classified as chloroplasts, mitochondrial, and non-bacterial were removed from the dataset. Using blanks and no-template controls, we identified 16 ASVs as contaminants using the R package decontam (45) and discarded these contaminants from the dataset (losing 0.06% of the reads). In addition, we removed ASVs represented by two or fewer reads in any given sample. After these filtering steps, mean read counts across all 132 samples was approximately 14,000 reads per sample. The mean read counts of DNA extraction blanks and no-template controls were 622 and 161, respectively. For specific analyses (details below), data were rarefied to 4,000 reads per sample.

### Statistical analyses

Statistical analyses were conducted using the R package vegan (46). The R code can be found in the supplemental information (Supplemental R code 1). Results were considered significant at a *P*-value < 0.05. The core ASVs were determined using the method based on abundance-occupancy distributions (47) on the rarefied dataset (Supplemental R code 2) with the assumption that the consistency of core ASVs in bacterial communities implies an important role of these ASVs in host plant fitness (47). Alpha diversity, observed ASV richness, and the inverse Simpson index were also determined using the rarefied dataset. Differences between means were tested using the Wilcox test function as data were not normally distributed. Correlations between factors were determined using the Kendall rank correlation test.

Beta dissimilarities between samples were determined as Bray-Curtis dissimilarities based on ASV relative abundances within samples. We used PERMANOVA models (adonis2 function) to identify factors explaining variation in the Bray-Curtis dissimilarities. In these models, the order of the variables will determine the importance of factors, with the first factor absorbing variation from subsequent factors. Therefore, sample characteristics entered the model in order of specificity, with the most specific characteristics (e.g., stomatal density) first and the factors encompassing more general characteristics (e.g., host plant species) later. Plant individual was considered as nested in host plant species.

The neutral models for occupancy-abundance curves were determined for all samples together and for upper and lower leaf surface samples separately. The R code from Burns et al. (48) was used to fit the neutral model from Sloan et al. (49) (Supplemental R code 2).

To determine which taxa were correlated with pH, stomatal density, and leaf side, we conducted differential abundance analyses using the R packages DESeq2 (50) and phyloseq (51). Due to the requirement of the original read count data in this analysis, we started from the unrarefied dataset where ASVs represented by one or two reads per sample were not discarded but ASVs that had a total relative abundance of less than 0.1% were discarded. For the continuous variables, stomatal density, and pH, we did an analysis using the likelihood ratio test (LRT) of the DESeq function. For leaf sides, we did a Wald significance test of the DESeq function.

The degree of endemism of each ASV was determined, after discarding ASVs that occurred in no more than one sample. Here, as a proxy for the degree of endemism of an ASV, we determined the proportion of plant species on which it was not detected. Weighted endemism of samples was then calculated as the product of the relative abundance of an ASV and its degree of endemism. We log-transformed weighted endemism to analyze normally distributed data. We then optimized a multiple regression model including pH, leaf side, and stomatal density as explanatory variables for the weighted endemism of samples. We used a paired Wilcoxon test to assess differences in endemism between upper and lower surfaces of leaves.

## RESULTS

**Fig 1:**
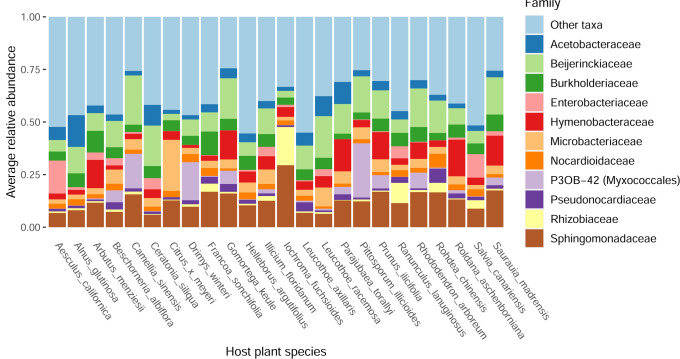
Barplot representing the relative abundance of the 11 most abundant bacterial genera. Each bar represents the average relative abundance in samples of a given plant species (generally *n* = 6, representing upper and lower leaf surface samples from three individual plants).

We used 16S rRNA gene sequencing to characterize the phyllosphere communities found on 132 leaf samples representing 24 different plant species, with all plant species sampled in the University of California Botanical Garden at Berkeley (which is approximately 0.14 km^2^). Recorded plant and leaf characteristics can be found in Supplemental Table 1. After quality filtering of the 16S rRNA reads (see Materials and Methods), we obtained a total of 8,584 ASVs representing 1,185 bacterial genera. The most abundant genera were *Sphingomonas* (11.5%), *Methylobacterium* (7.6%), and *Hymenobacter* (6.3%). The abundances of the 11 most abundant genera are visualized in Fig. 1. Additionally, core ASVs were determined using the occupancy-abundance approach developed by Shade and Stopnisek (47). These core ASVs are bacteria which are hypothesized to have a functional relationship with the plant host because of their high occurrence in the samples and contribution to beta diversity between the samples (47). Despite having sampled the phyllosphere of 24 different plant species, this analysis uncovered a cross-plant-species core microbiome that encompassed 56 ASVs which represented 38% of all reads (Supplemental Table 2).

A Mantel test comparing the geographic distance between individual plants to the Bray-Curtis dissimilarities in phyllosphere bacterial communities was not significant (Spearman rank *P*-value = 0.23), indicating that there is no distance-decay relationship for the phyllosphere communities across the sampling area.

When the compositions of the phyllosphere communities were compared using Bray-Curtis dissimilarity analyses, an overall PERMANOVA model (including all samples) shows that the variation in microbiome composition is explained by, in order of importance: host plant species, plant individual/leaf sample, leaf side by host plant species interaction, leaf side, stomatal density, and leaf surface pH (Table 1). The strong interaction effect observed between leaf side and plant species (Table 1; *R^2^* = 0.12; Supplemental Figure 1) motivated us to further analyze the upper and lower leaf samples separately given that, even within a given plant species, the two leaf sides appear to harbor distinct bacterial communities.

**TABLE 1:** *R^2^* values for leaf and plant characteristics of interest explaining Bray-Curtis dissimilarity variation between samples in a PERMANOVA model (9,999 permutations)^*a*^

Factor	*R* ^2^
Leaf surface pH	0.01**
Stomatal density	0.02***
Leaf side (upper/lower)	0.01***
Host plant species	0.32***
Plant individual	0.32***
Interaction of side and host plant species	0.12***

^*a*^ Factors are in the order they were added to the model. The full model for all variables examined can be found in Supplemental Table 3. *P-value < 0.05, **P-value < 0.01, ***P-value < 0.001.

**Fig 2:**
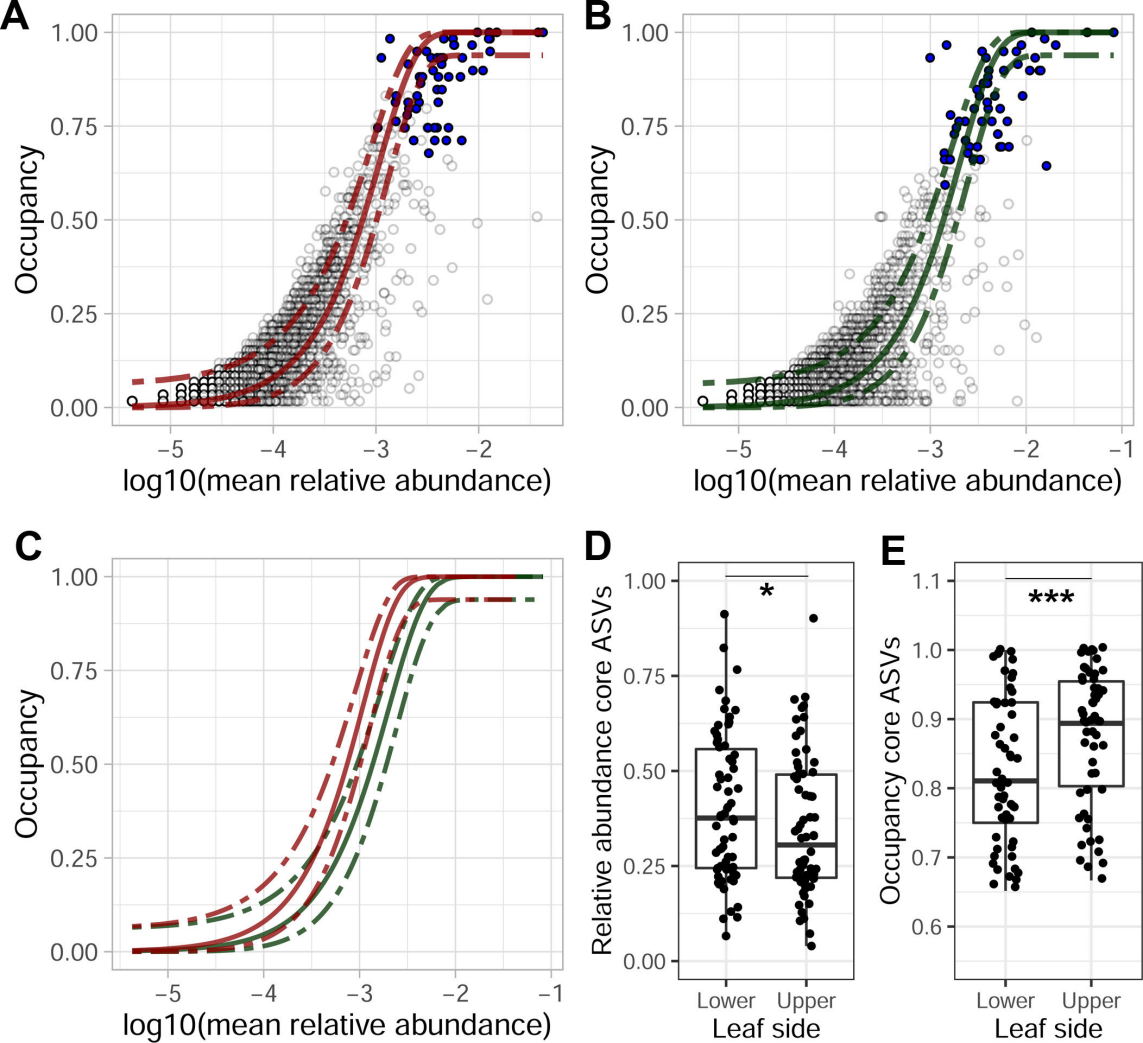
Occupancy-abundance curves of ASVs in (**A**) the upper leaf surface samples only and (**B**) the lower leaf samples only. Each circle represents the observations of an ASV, with the blue dots representing the observations of core ASVs. The lines in each graph represent the neutral model based on the upper samples (red) and lower samples (green), which are directly compared in (**C**). The (**D**) relative abundance and (**E**) occupancy of core ASVs for each of the leaf sides differed significantly (one-sided paired Wilcoxon test) with *P-value < 0.05, **P-value < 0.01, and ***P-value < 0.001.

We compared community characteristics between upper and lower leaf surfaces separately. The occupancy-abundance curve was a closer fit to the neutral model (*R^2^* = 0.72) for the upper leaf surface samples as compared to the fit for lower leaf surface samples (*R^2^* = 0.61, Fig. 2A and B). Furthermore, core ASVs were typically more abundant on the lower leaf sides, whereas their occupancy (ubiquity across samples) was higher on the upper leaf sides (Fig. 2D and E).

**Fig 3:**
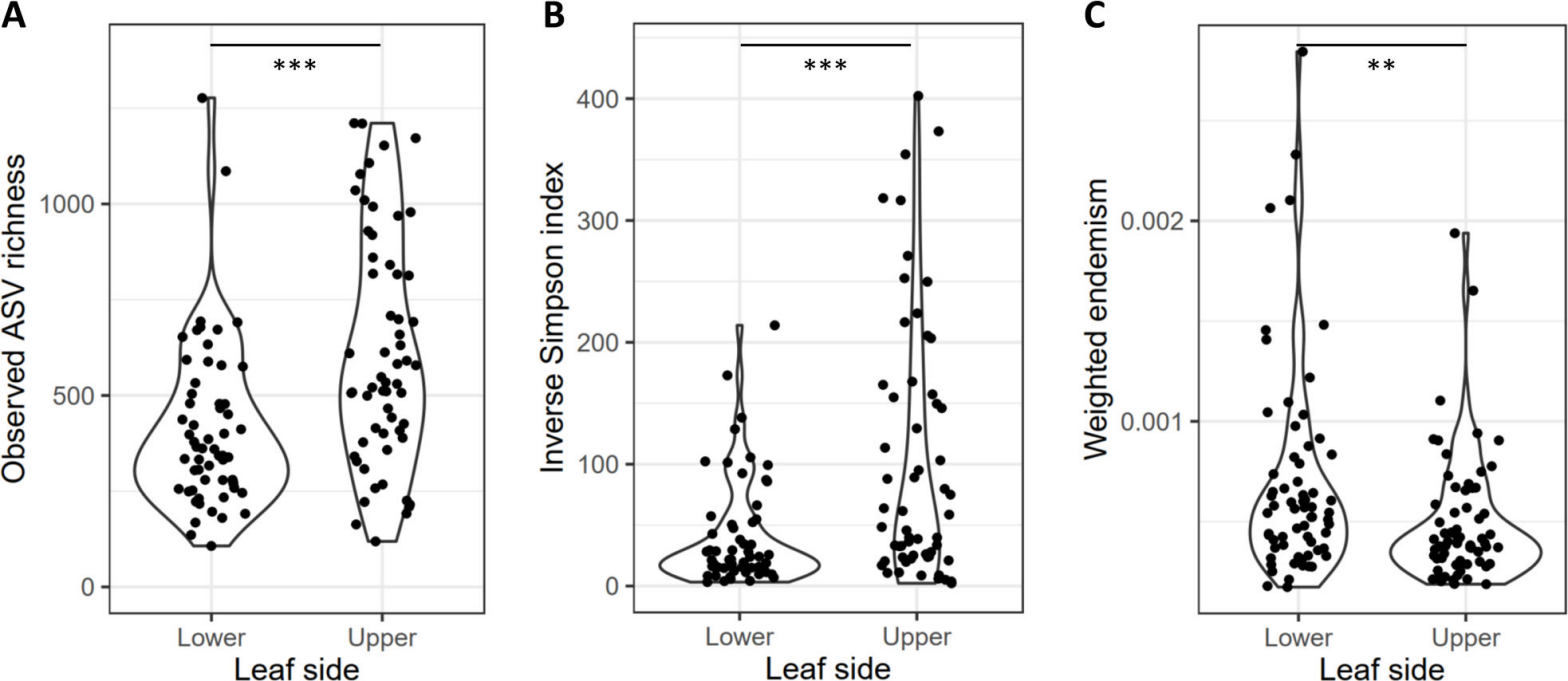
(**A**) The observed ASV richness and (**B**) the inverse Simpson index were determined per sample and showed significantly lower alpha diversities for lower leaf surfaces than for upper leaf surfaces. (**C**) The weighted degree of endemism for samples of each leaf side with *P-value < 0.05, **P-value < 0.01, and ***P-value < 0.001.

Both ASV richness and Simpson’s diversity were higher in the upper leaf surface phyllosphere communities than in the lower leaf surfaces (Fig. 3A and B). The weighted degree of endemism, an indicator of how restricted ASVs of a sample are to one or several host plant species compared to all host plant species sampled, was significantly higher for the lower leaf surfaces than for the upper leaf surfaces (paired Wilcoxon *P*- value = 0.005; Fig. 3C). This indicates a higher relative abundance of endemic ASVs on the lower surfaces of leaves than on the upper surfaces.

**Fig 4:**
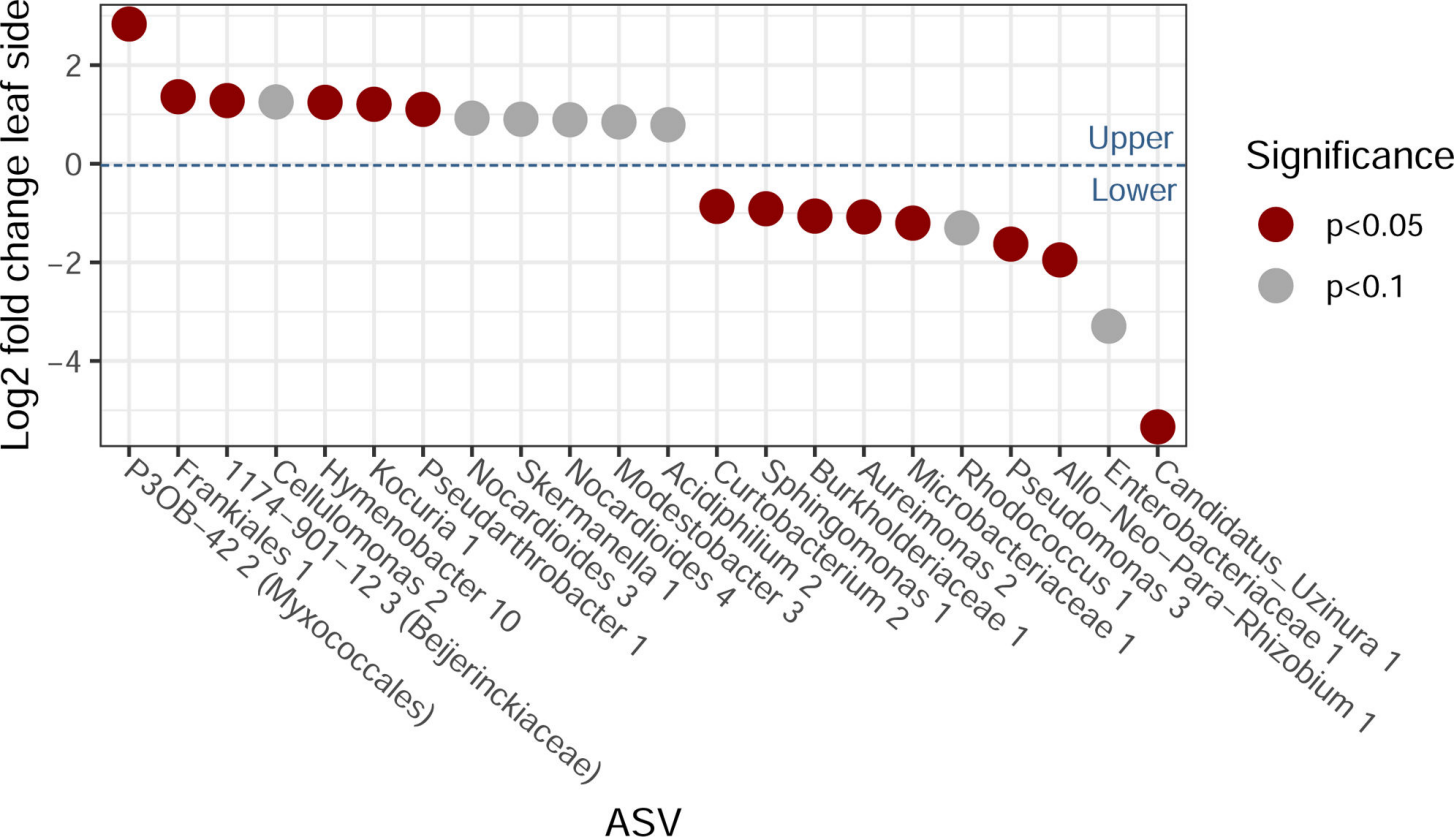
Significant and near-significant differentially abundant taxa between upper and lower surface communities of leaves. A positive log2 fold change indicates a preference for the upper sides of the leaves, and a negative log2 fold change indicates a preference for the lower sides of the leaves.

A differential abundance analysis of ASVs by leaf side identified several taxa that were relatively more abundant on the upper or lower leaf surfaces (Fig. 4). We found that 75% of the ASVs significantly associated with the lower leaf surfaces belonged to the core phyllosphere microbiome found in this study (Supplemental Table 2), whereas only 17% of the ASVs significantly associated with the upper leaf surface belonged to the core microbiome. Moreover, core ASVs were found to occupy a lower proportion of ASVs overall but had higher relative abundances on the lower leaf surface relative to the upper leaf surface (paired Wilcoxon *P*-value = 0.046 and *P*-value < 0.001, respectively; Fig. 2D and E).

**Fig 5:**
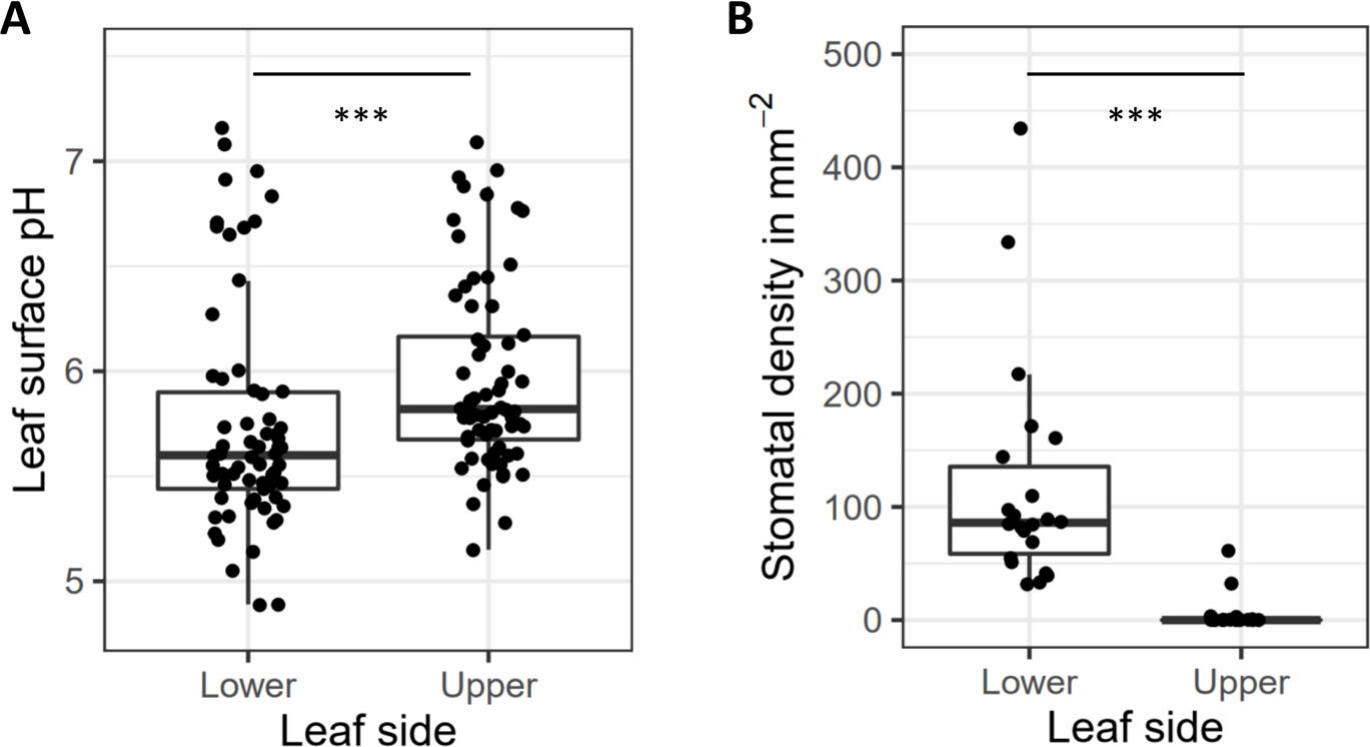
Boxplots showing mean and range of (**A**) pH and (**B**) stomatal density for lower and upper leaf sides separately (***P-value < 0.001).

Stomatal densities differed markedly between the upper and lower sides of the leaves of all species, as few or no stomata were found within the observed areas of the upper leaf surfaces of most plant species (Fig. 5B). In the case of the lower leaf surface, where most stomata were found, stomatal density is negatively, but not significantly, correlated with pH (*P*-value = 0.14, τ = −0.23). We did, however, observe a significant negative correlation between stomatal density and ASV richness (*P*-value = 0.003, τ = −0.27) across the lower leaf surface samples. A differential abundance analysis considering only the lower leaf surface samples revealed several ASVs that were significantly associated with stomatal density (Supplemental Figure 2). We found that 40% of the resulting ASVs associated with high numbers of stomata belonged to the core microbiome that we identified (Supplemental Table 2), whereas none of the low-stomata-associated ASVs belonged to the core microbiome.

The leaf surface pH measured in this study ranged from 4.89 to 7.16. The most important leaf characteristic associated with pH was the leaf side, with the lower leaf surfaces being 0.21 pH units more acidic, on average, than the upper surfaces (Fig. 5A). Leaf pH values did not differ significantly across plant species because the range of observed pH measurements of a given species was often quite high (Supplemental Figure 4). We found a significant correlation between pH and the observed ASV richness of the samples (*P*-value = 0.044, Kendall rank coefficient τ = 0.13), with higher—more neutral—pH values associated with higher bacterial richness, and a differential abundance analysis using DESeq2 revealed that the abundances of several bacterial ASVs were significantly correlated with pH (Supplemental Figure 3).

## DISCUSSION

This “common garden” study of the phyllosphere microbiome across 24 plant species revealed that, even though community composition of both sides of a leaf is rather similar, the relative impact of dispersal versus host plant selection on the phyllosphere microbiome differs between the upper and lower leaf surface. This may explain why previous phyllosphere studies show a discrepancy between the relative contribution of the environment or location versus host plant selection.

Our results show that plant species identity is the most important factor in this study driving community composition, confirming results of the previous studies (12, 20, 22, 52). When zooming in from the landscape to the single-leaf level, however, we find remarkable differences in microbial community structure driven by the leaf side. The distinction between upper and lower leaf surfaces was characterized by a strong interaction effect between plant species and leaf sides (Table 1). The occupancy-abundance neutral models differed between upper and lower leaf surfaces, and we observed a shift in the average occupancy-abundance relationship (Fig. 2C) that indicated taxon-specific occupancy was higher on the upper leaf surfaces compared to the lower leaf surfaces. This supports the hypothesis that the upper leaf surfaces are more likely to accumulate dispersing microbes than the lower leaf surfaces. In contrast, the fit of the neutral model was higher for the upper leaf surfaces than for the lower leaf surfaces (Fig. 2A and B), suggesting that neutral processes such as dispersal, drift, and the composition of the metacommunity might be more important in shaping these communities. As we detected no distance-decay relationship, we considered the impact of variation in the metacommunity to be negligible at the scale of this study. Hence the difference in fit to the neutral model likely indicates that either dispersal, drift, or both of these processes are relatively more important in upper versus lower leaf surfaces. This aligns with our hypotheses of relatively more dispersal and also a greater importance of drought and UV stress on the upper leaf surfaces compared to the lower leaf surfaces. It is important to note that fitting neutral models have some limitations (53), and the patterns we observe to be typical for the neutral model could be the result of other processes (54). Nevertheless, multiple lines of evidence support our hypotheses. First, as core ASVs likely have greater contributions to host interactions (47), it is noteworthy that core ASVs were found to be both more abundant on lower leaf surfaces (Fig. 2D) and more often associated with the lower leaf surfaces (Fig. 4). Likewise, we observed higher numbers and abundances of more endemic ASVs on the lower leaf surfaces (Fig. 3C), which suggests that dispersal becomes relatively less important in relation to selection pressure on lower leaf surfaces as compared to upper leaf surfaces. To summarize, we found lower richness (Fig. 3A and B), higher abundances of core ASVs (Fig. 2D and Fig. 4), and more endemic ASVs on lower leaf surfaces (Fig. 3C), indicating that lower leaf surfaces act as a relatively more selective, plant-associated environment than the upper leaf surfaces. Focusing on this top-bottom dichotomy is likely to yield observations that are more informative of the role of both the plant features driving phyllosphere microbial community assembly and conversely the role of microbiomes in shaping plant functional traits (12) and ecosystem functions (55).

Within this leaf side framework, our study illustrates how bacterial community structure is not only predicted by leaf side itself, but by the varying topographic (i.e., stomatal density) and chemical (i.e., surface pH) characteristics distinguished between upper and lower leaf surfaces. Leaf surface pH was significantly correlated with leaf microbial community richness. Similar to previous results from soil and wetland environments (56, 57), leaf surface community richness was found to increase as pH shifted toward more neutral conditions, with several taxa being significantly correlated with pH (Supplemental Figure 3). These results are perhaps unsurprising given that distinct bacterial lineages can have distinct conservation of microbial pH preference (58) and a strong effect of pH on microbial metabolism (59). However, it is notable that the impact of pH was significant in this study despite a limited observed pH range (pH 4.9–7.2; Fig. 5A) compared to previous phyllosphere pH observations: pH 1 (carnivorous plants) – pH 11 (Malvaceae) (60). Interestingly, top sides of leaves were significantly more pH neutral, which may allow more bacterial taxa, including non-host specific taxa, to colonize the upper leaf surfaces.

Stomata are a critical leaf trait regulating processes such as gas exchange, transpiration, and microbial defense (61). In relation to the leaf surface microbiome, studies have shown stomatal grooves aid in microbial retention during chemical disturbance (62) and to a lesser degree leaf surface desiccation (10). In line with previous observations, we find stomata to exist almost exclusively on the lower leaf surfaces (Fig. 5B), where stomatal density was found to be negatively correlated with microbial diversity. Although further work is required to delineate the cause of this pattern, we speculate that stomata act as a component of plant filtering in the phyllosphere, which may act as micro-refugia or regulators of the micro-environment.

This study focused on the phyllosphere microbiome in relation to stomatal density and leaf surface pH, but the wealth of additional leaf traits that were not included opens up important questions about the role of other aspects of plant physiology in shaping host filtering among sites. Leaf traits across plant species tend to be well-correlated (63), and this “spectrum of leaf economics” ranges from slow to fast return on investments of nutrients (e.g., high leaf mass per area versus high photosynthetic capacity, respectively) (63), which itself has been shown to correlate with bacterial phyllosphere composition (12). In particular, increasing stomatal density is correlated with higher leaf thickness and lower chlorophyll content and hence is generally expected in plants with a slower return on nutrient investment (64, 65). Studies on leaf surface pH, however, are currently very limited, and it remains unclear how this trait might relate to others or if and how leaf pH is regulated by plants, especially in relation to the impact on leaf-associated microbial communities.

Overall, despite considerable diversity in leaf morphology and chemistry across plant species, phyllosphere microbial community structure at the single-leaf level has been traditionally overlooked in favor of macro-landscape level studies. The results of this study are in line with our hypothesis that leaf sides differ in phyllosphere microbiome assembly as a result of a different balance of host-plant selection versus neutral processes of immigration and differences in environmental stresses associated with desiccation and UV radiation on the upper versus lower leaf. These results suggest that future studies examining the impact of host selection on the leaf microbiome should particularly focus on the lower leaf side, where conditions are less variable and the communities are seemingly more impacted by plant host-imposed selection.

## Data Availability

All sequences are deposited under project accession number PRJEB55637.

## SUPPLEMENTAL MATERIAL

Supplemental code 1 mbio.01111-23-s0001.txt All R code used to test hypotheses and generate figures for the manuscript.

Supplemental code 2 mbio.01111-23-s0002.txt R code adapted from Shade and Stopnisek (2019) to determine core ASVs.

Supplemental Figures and Tables mbio.01111-23-s0003.docx Figures S1 to S4 and Tables S1 to S3.

ASM does not own the copyrights to Supplemental Material that may be linked to, or accessed through, an article. The authors have granted ASM a non-exclusive, world-wide license to publish the Supplemental Material files. Please contact the corresponding author directly for reuse.
