# Supplementary material for: Leaf side determines the relative importance of dispersal versus host filtering in the phyllosphere microbiome
Source: mBio. 2023 Jul 12;14(4):e01111-23. doi: 10.1128/mbio.01111-23 (PMC10470611; doi:10.1128/mbio.01111-23)
Supplement: Supplemental Figures and Tables — Figures S1 to S4 and Tables S1 to S3. [file mbio.01111-23-s0003.docx]

Supplemental material


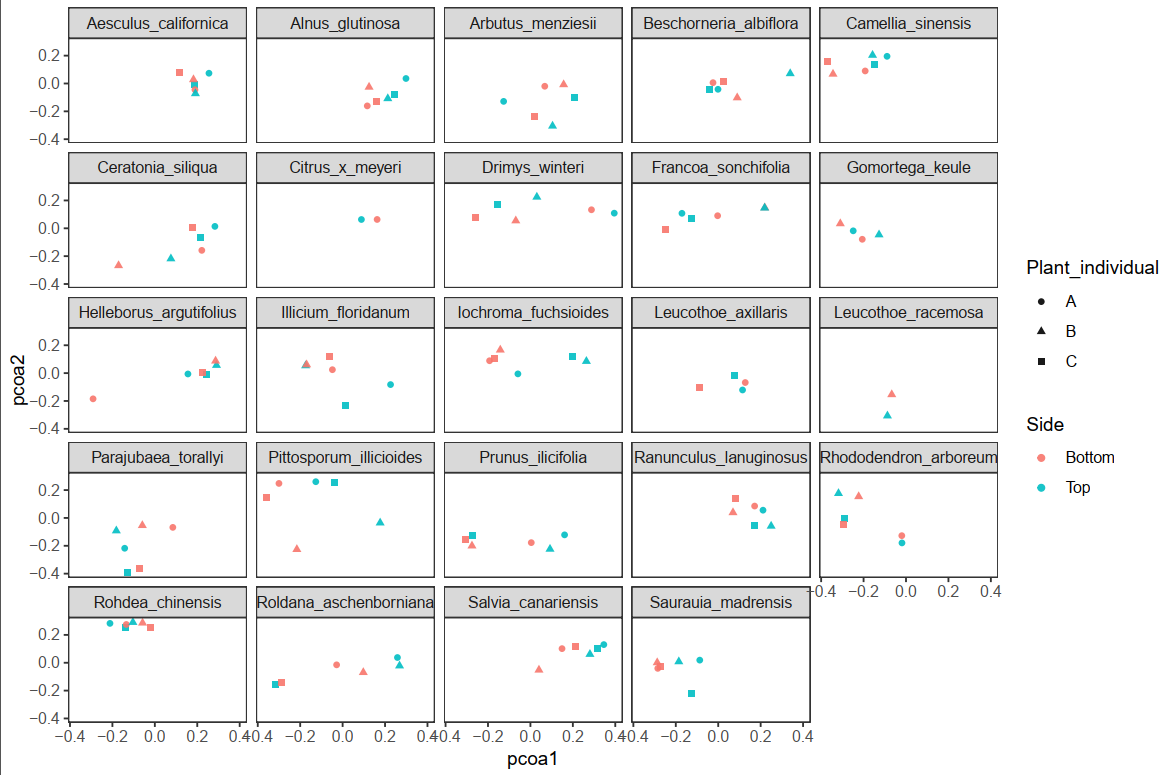


**Supplemental Figure 1:** Principal coordinate analysis (PCoA) of all samples, with samples represented per plant species in each panel.


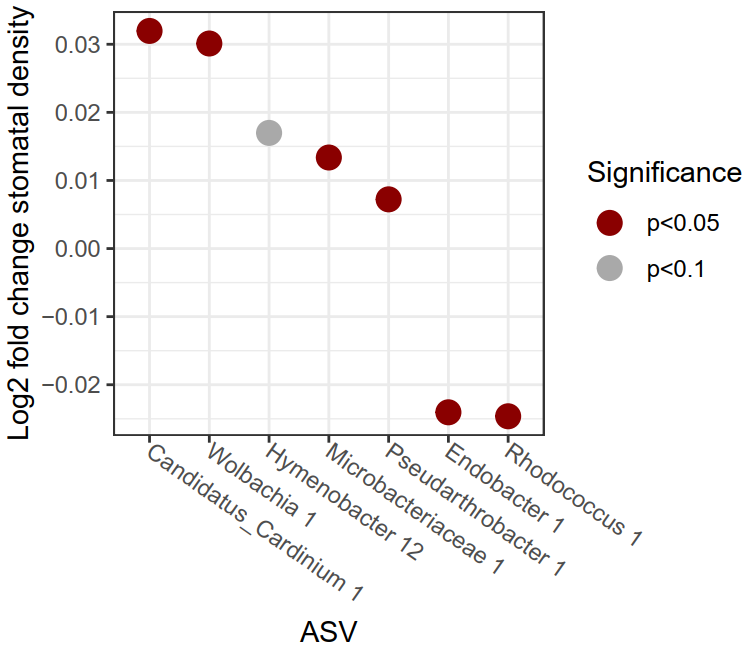


**Supplemental Figure 2:** ASVs that linearly correlated with stomatal density (mm^-2^) on the lower leaf surface.


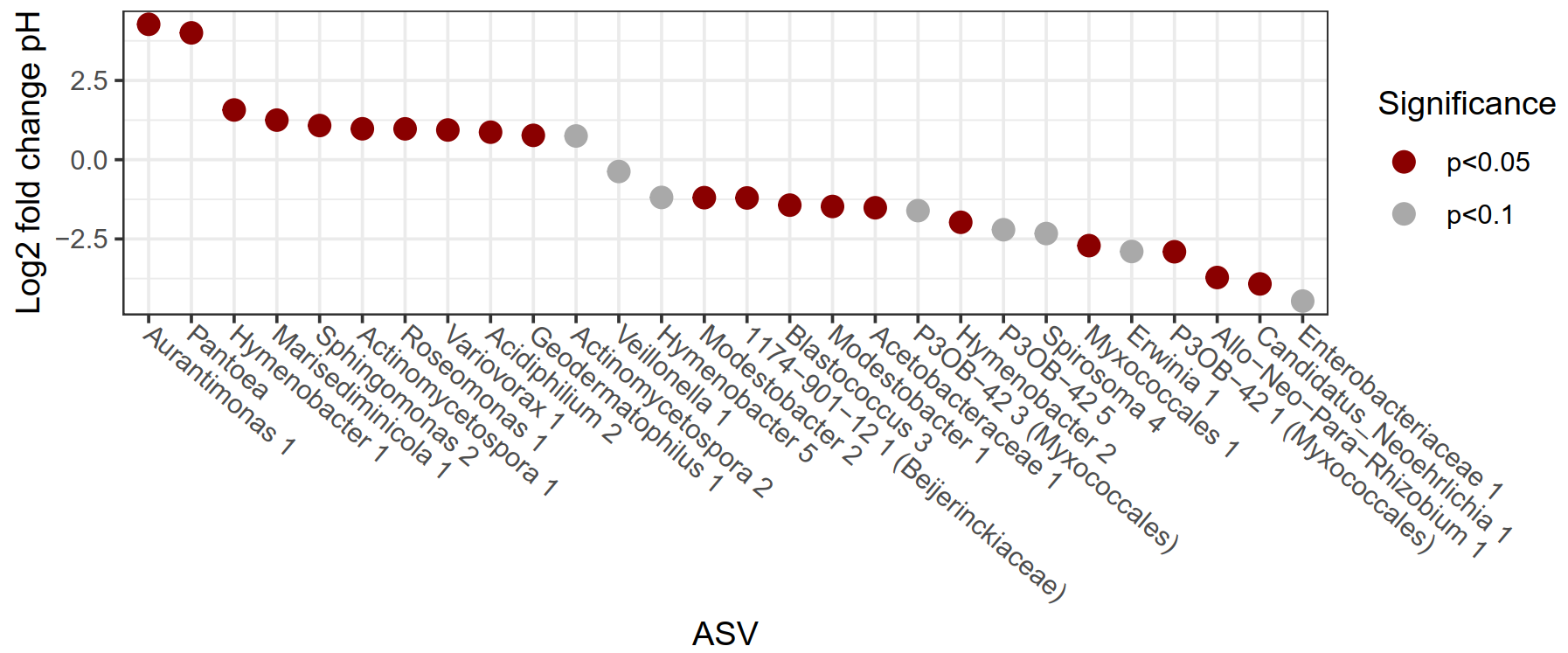


**Supplemental Figure 3:** ASVs that correlated with the leaf surface pH. Enrichment of taxa on more acidic leaf surface is represented by a negative Log2 fold change while a positive log2 fold change indicates a higher abundance of bacterial taxa on on a more neutral leaf surface.


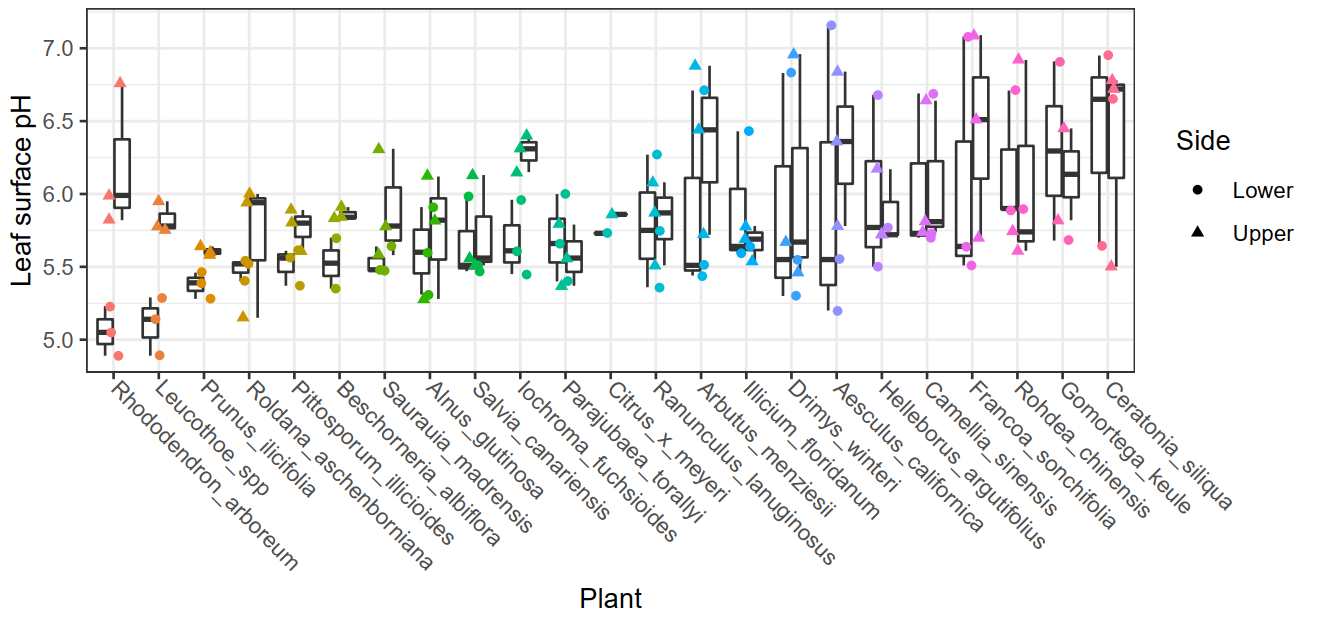


**Supplemental Figure 4:** The pH values measured (ordinate) for each plant species (abscissa). Separate boxplots are shown for each leaf side of a given plant species. Circles represent lower leaf surfaces, triangles upper leaf surfaces.

**Supplemental Table 1:** Host plant species sampled and their recorded characteristics.

| **Plant species** | **Growth form** | **Evergr./decid.** | **Region of native origin** | **Leaf side** | **Stomatal density (mm^-2^)** | **Mean pH** |
| --- | --- | --- | --- | --- | --- | --- |
| Aesculus californica | Tree-like | Deciduous | California | Upper | 0 | 6.3 |
|  |  |  |  | Lower | 92 | 6 |
| Alnus glutinosa | Tree-like | Deciduous | Mediterranean | Upper | 0 | 5.7 |
|  |  |  |  | Lower | 39 | 5.6 |
| Arbutus menziesii | Tree-like | Deciduous | California | Upper | 0 | 6.3 |
|  |  |  |  | Lower | 89 | 5.9 |
| Beschorneria albiflora | Shrub | Evergreen | Central America | Upper | 61 | 5.9 |
|  |  |  |  | Lower | 33 | 5.5 |
| Camellia sinensis | Shrub | Evergreen | Asia | Upper | 0 | 6.1 |
|  |  |  |  | Lower | 110 | 6 |
| Ceratonia siliqua | Tree-like | Evergreen | Mediterranean | Upper | 0 | 6.3 |
|  |  |  |  | Lower | 82 | 6.4 |
| Citrus x meyeri | Tree-like | Evergreen | Asia | Lower | 434 | 5.7 |
|  |  |  |  | Upper | 0 | 5.9 |
| Drimys winteri | Tree-like | Evergreen | South America | Upper | 0 | 6 |
|  |  |  |  | Lower | 84 | 5.9 |
| Francoa sonchifolia | Herbaceous perennial | Evergreen | South America | Lower | 55 | 6.1 |
|  |  |  |  | Upper | 1 | 6.4 |
| Gomortega keule | Tree-like | Evergreen | South America | Lower | 87 | 6.3 |
|  |  |  |  | Upper | 0 | 6.1 |
| Helleborus argutifolius | Herbaceous perennial | Evergreen | Mediterranean | Upper | 0 | 5.9 |
|  |  |  |  | Lower | 69 | 6 |
| Illicium floridanum | Shrub | Evergreen | Central America | Upper | 3 | 5.7 |
|  |  |  |  | Lower | 41 | 5.9 |
| Iochroma fuchsioides | Shrub | Evergreen | South America | Upper | 0 | 6.3 |
|  |  |  |  | Lower | 97 | 5.7 |
| Leucothoe axillaris | Shrub | Evergreen | Eastern North America | Lower | 79 | 5.2 |
|  |  |  |  | Upper | 0 | 5.8 |
| Leucothoe racemosa | Shrub | Deciduous | Eastern North America | Lower | NA | 4.9 |
|  |  |  |  | Upper | NA | 5.8 |
| Parajubaea torallyi | Tree-like | Evergreen | South America | Lower | NA | 5.7 |
|  |  |  |  | Upper | NA | 5.6 |
| Pittosporum illicioides | Shrub | Evergreen | Asia | Lower | 171 | 5.5 |
|  |  |  |  | Upper | 0 | 5.8 |
| Prunus ilicifolia | Tree-like | Evergreen | California | Upper | 3 | 5.6 |
|  |  |  |  | Lower | 161 | 5.4 |
| Ranunculus lanuginosus | Herbaceous perennial | Deciduous | Mediterranean | Upper | 0 | 5.8 |
|  |  |  |  | Lower | 51 | 5.8 |
| Rhododendron arboreum | Shrub | Evergreen | Asia | Upper | 0 | 6.2 |
|  |  |  |  | Lower | 334 | 5.1 |
| Rohdea chinensis | Herbaceous perennial | Evergreen | Asia | Upper | 0 | 6.1 |
|  |  |  |  | Lower | 32 | 6.2 |
| Roldana aschenborniana | Herbaceous perennial | Evergreen | Central America | Upper | 0 | 5.7 |
|  |  |  |  | Lower | 85 | 5.5 |
| Salvia canariensis | Shrub | Evergreen | Mediterranean | Upper | 32 | 5.7 |
|  |  |  |  | Lower | 217 | 5.7 |
| Saurauia madrensis | Tree-like | Evergreen | Central America | Upper | 0 | 5.9 |
|  |  |  |  | Lower | 144 | 5.5 |

**Supplemental Table 2:** Core members of the phyllosphere microbiome on plants collected at the University of California Botanical Garden at Berkeley. The total relative abundance of an ASV in the dataset and the proportion of samples in which it was found are included.

| **Family** | **ASV name** | **ASV_ID** | **Tot. Rel. abun.** | **Rel.occurrence** |
| --- | --- | --- | --- | --- |
| Sphingomonadaceae | Sphingomonas 1 | ASV_8 | 0.062 | 1 |
| Beijerinckiaceae | Methylobacterium 1 | ASV_14 | 0.041 | 1 |
| Beijerinckiaceae | Methylobacterium 2 | ASV_35 | 0.015 | 0.99 |
| Microbacteriaceae | Microbacteriaceae 1 | ASV_38 | 0.014 | 0.97 |
| Beijerinckiaceae | 1174-901-12 1 | ASV_52 | 0.013 | 0.94 |
| Acetobacteraceae | Acidiphilium 1 | ASV_46 | 0.013 | 0.92 |
| Sphingomonadaceae | Sphingomonas 2 | ASV_39 | 0.012 | 1 |
| Burkholderiaceae | Variovorax 1 | ASV_37 | 0.012 | 0.94 |
| Rhizobiaceae | Aurantimonas 1 | ASV_26 | 0.009 | 0.68 |
| Burkholderiaceae | Massilia 1 | ASV_59 | 0.009 | 0.96 |
| Beijerinckiaceae | 1174-901-12 2 | ASV_64 | 0.009 | 0.97 |
| Sphingobacteriaceae | Mucilaginibacter 1 | ASV_56 | 0.008 | 0.91 |
| Hymenobacteraceae | Hymenobacter 1 | ASV_57 | 0.008 | 0.85 |
| Sphingomonadaceae | Sphingomonas 3 | ASV_41 | 0.007 | 0.84 |
| Microbacteriaceae | Curtobacterium 1 | ASV_78 | 0.007 | 0.95 |
| Pseudonocardiaceae | Actinomycetospora 1 | ASV_55 | 0.006 | 0.92 |
| Deinococcaceae | Deinococcus 3 | ASV_65 | 0.006 | 0.7 |
| Beijerinckiaceae | Methylobacterium 4 | ASV_79 | 0.006 | 0.96 |
| Sphingomonadaceae | Sphingomonas 4 | ASV_69 | 0.006 | 0.74 |
| Hymenobacteraceae | Hymenobacter 2 | ASV_92 | 0.005 | 0.72 |
| Azospirillaceae | Skermanella 1 | ASV_127 | 0.005 | 0.81 |
| Kineosporiaceae | Kineococcus 1 | ASV_98 | 0.005 | 0.82 |
| Pseudomonadaceae | Pseudomonas 3 | ASV_68 | 0.005 | 0.69 |
| Micrococcaceae | Pseudarthrobacter 1 | ASV_129 | 0.004 | 0.88 |
| Burkholderiaceae | Massilia 2 | ASV_141 | 0.004 | 0.9 |
| Burkholderiaceae | Burkholderiaceae 1 | ASV_95 | 0.004 | 0.74 |
| Sphingomonadaceae | Sphingomonas 5 | ASV_120 | 0.004 | 0.97 |
| Beijerinckiaceae | Beijerinckiaceae 1 | ASV_152 | 0.004 | 0.81 |
| Hymenobacteraceae | Hymenobacter 3 | ASV_99 | 0.004 | 0.77 |
| Microbacteriaceae | Amnibacterium 1 | ASV_107 | 0.004 | 0.9 |
| Kineosporiaceae | Kineococcus 2 | ASV_124 | 0.004 | 0.92 |
| Beijerinckiaceae | Methylobacterium 5 | ASV_104 | 0.004 | 0.92 |
| Acetobacteraceae | Acidiphilium 2 | ASV_114 | 0.004 | 0.86 |
| Rhodobacteraceae | Rubellimicrobium 1 | ASV_140 | 0.004 | 0.75 |
| Microbacteriaceae | Curtobacterium 2 | ASV_158 | 0.003 | 0.81 |
| Hymenobacteraceae | Hymenobacter 4 | ASV_111 | 0.003 | 0.72 |
| Burkholderiaceae | Noviherbaspirillum 1 | ASV_186 | 0.003 | 0.81 |
| Geodermatophilaceae | Geodermatophilus 1 | ASV_122 | 0.003 | 0.91 |
| Microbacteriaceae | Frondihabitans 1 | ASV_133 | 0.003 | 0.88 |
| Kineosporiaceae | Kineococcus 3 | ASV_130 | 0.003 | 0.72 |
| Microbacteriaceae | Amnibacterium 2 | ASV_189 | 0.003 | 0.9 |
| Pseudonocardiaceae | Actinomycetospora 2 | ASV_156 | 0.003 | 0.74 |
| Burkholderiaceae | Mitsuaria 1 | ASV_187 | 0.002 | 0.81 |
| Burkholderiaceae | Massilia 4 | ASV_213 | 0.002 | 0.81 |
| Acetobacteraceae | Roseomonas 1 | ASV_161 | 0.002 | 0.79 |
| Rhizobiaceae | Aureimonas 2 | ASV_168 | 0.002 | 0.73 |
| Geodermatophilaceae | Blastococcus 1 | ASV_286 | 0.002 | 0.73 |
| Geodermatophilaceae | Blastococcus 2 | ASV_239 | 0.002 | 0.71 |
| Intrasporangiaceae | Janibacter 1 | ASV_240 | 0.002 | 0.79 |
| Beijerinckiaceae | Methylobacterium 6 | ASV_194 | 0.002 | 0.71 |
| Nocardioidaceae | Nocardioides 2 | ASV_268 | 0.002 | 0.72 |
| Sphingomonadaceae | Sphingomonas 8 | ASV_228 | 0.002 | 0.78 |
| Sphingomonadaceae | Sphingomonas 9 | ASV_179 | 0.002 | 0.71 |
| Acetobacteraceae | Acidiphilium 3 | ASV_259 | 0.002 | 0.81 |
| Veillonellaceae | Veillonella 1 | ASV_296 | 0.001 | 0.97 |
| Neisseriaceae | Neisseria 1 | ASV_356 | 0.001 | 0.93 |

**Supplemental Table 3**: PERMANOVA of all factors included in this study.

| **Factor** | **R^2^** | **p-value** |
| --- | --- | --- |
| Leaf surface pH | 0.01 | 0.004 |
| Stomatal density | 0.02 | <0.001 |
| Leaf side (upper/lower) | 0.02 | <0.001 |
| Leaf pubescence | 0.04 | <0.001 |
| Leaf hardness | 0.03 | <0.001 |
| Evergreen or deciduous plant | 0.03 | <0.001 |
| Plant type (herb/shrub/tree) | 0.04 | <0.001 |
| Climate of native origin | 0.06 | <0.001 |
| Region of native origin | 0.11 | <0.001 |
| Host plant species | 0.10 | <0.001 |
| Date sampled | 0.03 | <0.001 |
| Plant individual / leaf sample | 0.21 | <0.001 |
| Interaction of pH and host plant species | 0.17 | <0.001 |
| Interaction of side and host plant species | 0.11 | 0.021 |
